# Cryopreservation and its effects on motility and gene expression patterns and fertilizing potential of bovine epididymal sperm

**DOI:** 10.1002/vms3.355

**Published:** 2020-09-18

**Authors:** Hassan Nazari, Ebrahim Ahmadi, Hamid Hosseini Fahraji, Azita Afzali, Najmeh Davoodian

**Affiliations:** ^1^ Research Institute of Animal Embryo Technology Shahrekord University Shahrekord Iran; ^2^ PhD Student of Animal Reproductive Biotechnology Faculty of Veterinary Medicine Shahrekord University Shahrekord Iran; ^3^ PhD Candidate of Reproductive Biology Faculty of Medical Sciences Tehran University of Medical Sciences Tehran Iran

**Keywords:** bovine, embryo, epididymal sperm, freezing, IVF

## Abstract

Despite encountering new challenges in using epididymal sperm recovered from cauda epididymides, this accessible and, in some species, worthwhile sample makes inevitable the further development of a suitable cryopreservation protocol. In this study, sperm was recovered from the epididymis of 4°C overnight stored slaughtered bulls' testes and the effects of cryopreservation on the bovine epididymal sperm motility (with CASA) and gene expression patterns (with quantitative Real time‐PCR) were evaluated. Moreover the fertilizing potential of cryopreserved epididymal sperm was used in in vitro fertilization (IVF). After freezing and thawing of epididymal sperm, total and slow progressive sperm motility, VCL, VAP, MAD, ALH and BCF were significantly decreased (*p* < .05), while in the parameters of fast progressive motility, VSL, LIN, WOB and STR there were not any significant variations in the frozen sperm compared to fresh (non‐frozen) counterpart. The assessment of abundance of transcripts encoding motility (TSSK6) and fertility (PRM1 and PRM2)‐related genes in epididymal sperm, showed that these transcripts were affected by freezing especially in slow progressive motility status (*p* < .01). Furthermore, cleavage and blastocyst rate did not present any significant differences between bovine embryos produced in vitro by fresh or frozen‐thawed epididymal sperm. It can be concluded that epididymal sperm has enough freezability after overnight testes storage, and cryopreservation could not affect the percentage of in vitro produced embryos in spite of the changes of relative abundance of some transcripts and direction progressive motility pattern of sperm.

## Graphical abstract

The recovery of the epididymal spermatozoa from dead animals, the cryopreservation and subsequent IVF are useful tools to rescue genetic material that otherwise would be lost, either from highly productive animals or from endangered species. This study was designed for development of suitable cryopreservation protocols for bovine epididymal sperm. We collected testes tissues from a local slaughterhouse and stored them overnight in the refrigerator. Semen was recovered from the epididymis and the effects of cryopreservation on the patterns of bovine epididymal sperm motility and gene expression were evaluated and finally, the fertilizing potential was tested by IVF.

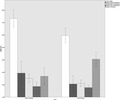

## 1 INTRODUCTION

The use of epididymal sperm as a valuable source of male gamete for assisted reproductive techniques (ART) has recently escalated. Sometimes, animal ART centres have easier and further access to this potential source in their researches and preserve the genetic ability of worth slaughtered animals or animals in the risk of extinction. Moreover this convenient source provides the conditions to do reproductive technologies such as artificial insemination (AI), IVF and so on. Also, applying the sperm cryopreservation technique allows a more economical and efficient way to use that material because it can be utilized anytime not just immediately after the death of an animal.

Some exclusive challenges arise when working with epididymal sperm. First, the majority of research has focused on increasing the effectiveness of cryopreservation and ART only utilizing ejaculated sperm. Second, epididymal and ejaculated sperm are different with regards to their exposure to seminal plasma. Epididymal sperm has been shown to have different respiration and metabolism rates, motility characteristics and plasma membrane integrity (Goovaerts et al., 2006; Miller, Winer, & Ax, 1990). The cauda epididymal sperm is less motile than ejaculated sperm and has also a lower velocity, straightness and linearity (Goovaerts et al., 2006). The epididymis of bull is very accessible and utilization of bovine as an animal model is expected to optimize the protocols of epididymal sperm cryopreservation for other species, specially human and endangered species.

Commonly, semen quality analysis has been considered as an initial option for fertility potential assessment. Visual parameters may not be adequate for a thorough evaluation of the fertility potential of semen. Several in vitro semen analysis methods have been proposed. Many laboratory techniques have been shown to confirm the fertility potential of a semen sample (Rodriguez‐Martinez, 2003, 2006; Rodriguez‐Martinez & Barth, 2007). Laboratory‐evaluated parameters of sperm quality include hyperactivated motility (Davis, Niswander, & Katz, 1992; Mortimer, 1990), sperm–oocyte interaction (Madrid‐Bury et al., 2005; Rodriguez‐Martinez, 2006) in addition to assessment of the integrity of various structures such as genomic DNA (gDNA), cell membrane, acrosome and mitochondria. In bovine cauda epididymal sperm cryopreservation, there are limited studies to the assessment of motility pattern of epididymal sperm with computer‐assisted sperm analyzer (CASA) and gene expression fluctuations of some important regulatory transcripts involved in motility and fertility and their relation to egg fertilizing potential of these type of sperm subsequent of cryopreservation.

Based on the finding of some studies (Selvaraju, Krishnan, Archana, & Ravindra, 2016; Selvaraju et al., 2018; Shilpa et al., 2017), occurred changes in transcript levels of some genes during cryopreservation need to be well‐defined to optimize cryopreservation and fertility procedures.

Serine/threonine testis‐specific protein kinase (TSSK6) was found to be associated with high‐motility status (Bissonnette, Lévesque‐Sergerie, Thibault, & Boissonneault, 2009) and gamete fusion (Sosnik et al., 2009) and both protamine 1 and 2 are related to fertility potential of sperm (Ganguly et al., 2013; Oliva, 2006) and/or early embryogenesis (protamine to histone exchange, DNA methylation, start of transcription; Hecht et al., 2011).

The aim of the present study was, therefore, evaluation of the effect of cryopreservation of epididymal sperm on the sperm motility and expression patterns of few selected genes related to motility (TSSK6) and fertility (PRM1 and PRM2) in epididymal sperm, and finally, find a relation between motility and gene expression patterns of evaluated genes and fertilizing potential in in vitro embryo production.

## 2 MATERIALS AND METHODS

### 2.1 Chemicals

Unless stated otherwise, chemicals were obtained from Sigma Chemical Co.

### 2.2 Ethics statement

Animal husbandry and handling were conducted by the guidelines of the ethical committee of Shahrekord University, Iran.

### 2.3 Sperm collection and motility assessment

To perform this study, randomly one testes of slaughtered crossbred bulls (*n* = 10), was removed and transferred to the laboratory within 2h at room temperature (19°C–22°C) and placed at 4°C overnight. In the lab, epididymides of testes were exposed by cutting the tunica vaginalis. Cauda epididymides were held with forceps, and multiple incisions were made in the tubuli with a bistoury. Sperm was collected with a blade and transferred to the Tris base medium (Tris: 200 mM, Citric acid: 66.7 mM, D‐Fructose: 55.5 mM) and kept at room temperature for 1 hr to receive the maximum motility.

The sperm motility was determined by computer‐assisted sperm analyzer (CASA; Hooshmand Fanavar) and those samples that had more than 50% motility, were used for experiments. Ten microlitre of sperm suspension was loaded on a pre‐warmed Spermmeter semen analysis chamber (Sperm Processor Pvt. Ltd.; 10 µm depth) and then evaluated. For each sample, six vision fields were analysed according to the following set of parameters: Frames acquired: 30 frame per second; low‐linear velocity (VSL) cut‐off: 5 µm s^−1^; high‐VSL cut‐off: 20 µm s^−1^. Magnifying power was ×4 for microscope objective lens. The motility descriptors obtained after CASA analyses were total motility including four motility classes (fast progressive [class A], slow progressive [class B], non‐progressive [class C] and immotile [class D], respectively; %), curvilinear velocity (VCL; µm s^−1^), linear velocity (VSL; µm s^−1^), mean velocity (VAP; µm s^−1^), mean angular displacement (MAD; degrees), mean amplitude of lateral head displacement (ALH; µm), frequency of head displacement (BCF; Hz), linearity coefficient (LIN; %); wobble coefficient (WOB; %) and straightness coefficient (STR; %).

### 2.4 Cryopreservation and thawing of sperm

The cauda epididymal sperm cells from each bull were diluted slowly to a final concentration of 50 × 10^6^ sperm/ml, using Tris egg‐yolk medium (73% Tris base medium, 20% egg‐yolk, and 7% glycerol; van Wagtendonk‐de Leeuw, Haring, Kaal‐Lansbergen, & den Daas, 2000). sperm was processed as described previously by Chaveiro, Machado, Frijters, Engel, & Woelders, (2006). Briefly, the diluted sperm was packed in 0.25 ml ‘french’ straws (IMV), at room temperature (24°C), and closed with a plug of polyvinylalcohol. The straws were placed horizontally in a water‐filled (24°C) box, in a place at 4°C for 105 min (slow cooling and equilibration period). The cooled straws were exposed to liquid nitrogen (LN2) vapour for 8 min (4 cm above LN2 level, approximately −125**°**C to −130**°**C), plunged into LN2, and stored in LN2 for at least one week. The experiment was designed with 10 replicates and each replicate was performed on a separate sample. For each sample, the cauda epididymal sperm is cryopreserved in at least three straws (for sperm motility and gene expression patterns evaluation, and IVF).

Thawing was achieved by immersing the straws for 6 s in a water bath set at 65°C (Eriksson & Rodriguez‐Martinez, 2000). Immediately after thawing, the sperm was processed for motility and molecular evaluations.

### 2.5 Molecular evaluation

#### 2.5.1 RNA extraction and cDNA synthesis

The molecular assessment was performed on high and low motile sperm that were separated by centrifugation of fresh (control group) or frozen‐thawed (treatment group) on a discontinuous Histoprep® (BAG Health Care) density gradient (0.5 ml 50% Histoprep over 0.5 ml 100% Histoprep) at 250 g for 7 min. After the centrifugation, the high motile sperm passed through the two gradients, while the low motile sperm passed only the 50% gradient. Total RNA was extracted from sperm using Iraizol reagent (RNA Biotechnology Company). Briefly, samples were lysed in 400 µl of this reagent and after 5 min mixed with 100 µl chloroform. The resulting mixture was centrifuged after 5 min (8000 *g*, 4°C, 5 min), yielding an upper aqueous phase containing total RNA. After addition of 500 µl absolute ethanol to the supernatant (20 min in −20°C), it was centrifuged (8000 *g*, 4°C, 5 min) and the RNA pellet was washed with 80% ethanol twice. The RNA samples were re‐suspended in 20 ml DEPC‐treated water and treated with RNase‐free DNase (Sinaclon Bioscience) to avoid amplification of contaminating genomic DNA. The amount and quality of RNA were determined by spectrophotometry (Amersham Pharmacia ultrospec 1100 Pro). Only RNA of sufficient purity, having an absorbance ratio (A260/280) greater than 1.9, was considered for the synthesis of cDNA.

Total RNA was reverse transcribed into cDNA in immediately after extraction (less than 2 hr) using M‐MLV reverse transcriptase (Sinaclon Bioscience) as described by Nazari, Shirazi, Shams‐Esfandabadi, Afzali & Ahmadi, (2016). The reverse transcription was done in a 20 µl volume containing 10 µl (14 µg) of extracted RNA and 1 µl random hexamer. This mixture was heated to 70°C for 5 min, and then 0.5 µl of RNase inhibitor, 2 µl RT buffer (50 mM Tris‐HCl, 75 mM KCl, 3 mM MgCl2), 2 µl dNTP (10 mM) and 1 µl M‐MLV reverse transcriptase were added. This mixture was incubated for 5 min at 25°C, followed by 60 min at 42°C. The mixture was heated to 70°C for 10 min to denature the RNA and then stored at −20°C.

#### 2.5.2 Quantitative real‐time PCR

Real‐time PCR was performed in two replicates for each sample (Rotor‐Gene Q 6000). The GenBank accession numbers, sequences, the size of amplified products and annealing temperature of each primer are shown in Table 1. Half ml DNase I treated cDNA (containing 0.2 µg) was added to 6 µl of SYBR Premix Ex Taq II Mix and 0.75 µM of each specific primer in a total volume of 12 µl. The PCR programme was comprised of 45 cycles of 94°C for 40 s, 60°C–63°C for 30 s (annealing temperature; Table 1) and 72°C for 30 s.

**TABLE 1:** Sequence and annealing temperature of used primers for RT‐PCR

Gene	Gen Bank accession no.	Primer sequence (5′−3′)	Product size (bp)	Annealing temp. (°C)
*GLUT5* (*SLC2A5*)	NM_001101042.2	F‐CCATCCTCCTCGGATTGACTG R‐CTCCTCTATCTCCGCATCCAC	170	63
*PRM1*	NM_174156.2	F‐CACCATGGCCAGATACCGATG R‐ CCTTATGACGGTGTAGCGACG	145	63
*PRM2*	NM_174157.4	F‐GACAGACCCGACCAACACTG R‐CCGGGTGGACTTTCAGTTGG	165	63
*TSSK6*	XM_588888.7	F‐CGAGGTACTGCTGGGCATTC R‐CGAGTCGTCGAAGGGCATAC	106	60

Considering the selection of an appropriate housekeeping gene as a reference gene for normalization, several studies demonstrated that the *Glut5* gene is highly reliable for analysis of relative gene expression in bovine fresh and frozen‐thawed sperm samples (Ashish et al., 2017). Melt curve analysis was conducted to confirm the specificity of each product. The no‐template control and no‐reverse transcriptase control were considered to check contamination of the PCR reagents. Data were analysed using LinReg PCR software version 2012.0 (USA), to give the threshold cycle number (Ct). Mean efficiency values (E) for each gene were also determined from the amplification profiles of individual samples using the same software (Ruijter et al., 2009). The following formula was applied to determine the relative gene expression in cloned embryos compared to the control group (IVF embryos; Dorak, 2007; Pfaffl, 2001).

$$Ratio=\frac{E_{GLUT5}\left(C_{t}\;frozen\;thawed\;sperm\right)/E_{\mathrm{target}}\left(C_{t}\;frozen\;thawed\;sperm\right)}{E_{GLUT5}\left(C_{t}I\;fresh\;sperm\right)/E_{\mathrm{target}}\left(C_{t}\;fresh\;sperm\right)}$$

### 2.6 In vitro embryo production

Bovine in vitro fertilization derived embryos were produced as described previously (Shirazi, Nazari, Ahmadi, Heidari, & Shams‐Esfandabadi, 2009). In brief, the in vitro matured oocytes in TCM199 supplemented with 10% FBS and 0.1 IU/ml FSH, were exposed to motile spermatozoa in two groups, the fresh and frozen‐thawed epididymal sperm prepared by centrifugation on a discontinuous histoprep density gradient (50% and 100%) at 250 g for 7 min (Ahmadi, Nazari, & Hossini‐Fahraji, 2019). During IVF, each 10 oocytes were cultured in a 50 µl droplet of TALP medium supplemented with 6 mg/ml bovine serum albumin (BSA) and incubated with 1 × 10^6^ motile spermatozoa for 22–24 hr at 39⁰C in 5% CO2. After fertilization, presumptive zygotes were mechanically denuded and cultured in SOFaaBSA medium in 5% CO2, 7% O2 and 88% N2. Cleaved embryos were separated 48 hr after in vitro culture (IVC) and were cultured in SOFaaBSA supplemented with 5% charcoal stripped FBS. The rate of blastocyst formation was recorded on days 7 and 8 after IVF. The cleavage and blastocyst rates are defined as the proportion of cleaved embryos and produced blastocysts per presumptive zygotes, respectively.

### 2.7 Statistical analysis

The mean ± *SEM* differences in motility pattern and embryonic developmental rate between fresh and frozen‐thawed sperm were analysed using independent samples *t* test. The relative abundance of gene expression between low and high motile sperm in fresh and frozen‐thawed sperm groups was analysed using one‐way analysis of variance (ANOVA) followed by Tukey post hoc test. All of the statistical analysis was performed by SPSS 20.0.0 software (IBM Corp.). Differences were considered significant at *p* < .05.

## 3 RESULTS

**FIGURE 1:**
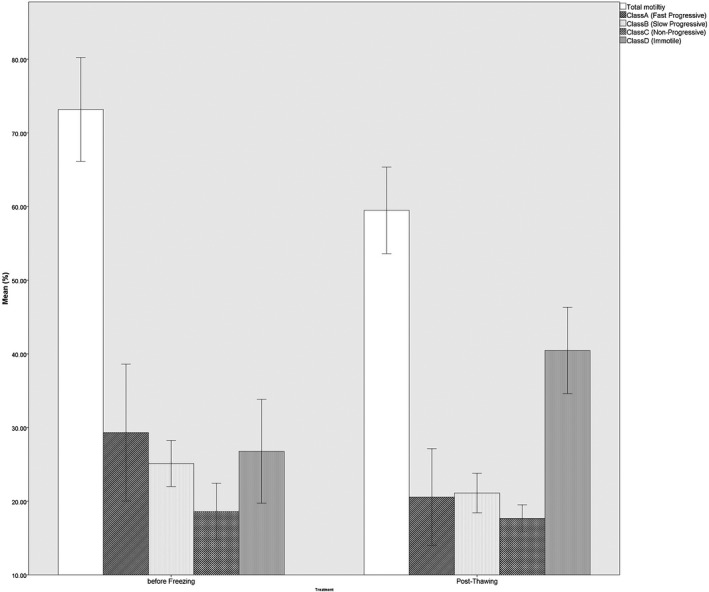
Percent motility (mean ± *SEM*) of prefreezed and post‐thawed bovine epididymal sperm. ^a,b^Refers to significant differences in the similar pattern columns(*p* < .05). Data represent *n* = 10 replicates

After cryopreservation and thawing of epididymal sperm, total motility of sperm and also slow progressive motility (class B; Figure 1), VCL, VAP, MAD, ALH and BCF (Table 2) was significantly decreased (*p* < .05) in fresh sperm (control group) compared to frozen‐thawed counterpart (treatment group), while the parameters of fast progressive motility (class A; Figure 1), VSL, LIN, WOB and STR (Table 2) did not have significant variations in the frozen‐thawed sperm compared to fresh.

**TABLE 2:** Effect of freezing on the bovine epididymal sperm motility patterns using CASA in vitro

	VCL (µm/sec)	VSL (µm/sec)	VAP (µm/sec)	MAD (°)	ALH (µm)	BCF (Hz)	LIN (%)	WOB (%)	STR (%)
Before freezing	78.64 ± 6.76^a^	31.86 ± 3.92	41.11 ± 4.25^a^	32.68 ± 3.98^a^	3.79 ± 0.22^a^	0.74 ± 0.12^a^	35.58 ± 2.23	50.07 ± 1.72	62.50 ± 2.63
Post‐thawing	52.97 ± 4.24^b^	22.26 ± 2.88	29.11 ± 3.07^b^	17.63 ± 2.00^b^	2.87 ± 0.14^b^	0.39 ± 0.07^b^	33.91 ± 1.85	48.90 ± 1.80	55.83 ± 2.32

Note

Abbreviations: ALH, amplitude of lateral head displacement; BCF; beat cross frequency; LIN: linearity; MAD, mean angular displacement; STR: straight line rate; VAP, average path velocity; VCL, curvilinear velocity; VSL, straight line velocity; WOB: wobble.

^a,b^Different superscripts in the same column denote a significant difference (*p* < .05). Data represent *n* = 10 replicates.

**FIGURE 2:**
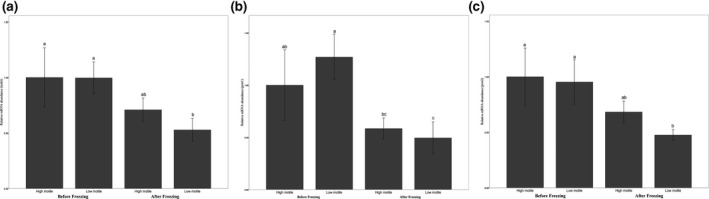
Relative abundance of *TSSK6* (A), *PRM1* (B) and *PRM2* (C) transcripts in bovine epididymal sperm derived overnight 4°C storage testes before and after freezing/thawing. mRNA was reverse transcribed, and subjected to real‐time quantitative PCR. All reactions were normalized for glut5 mRNA expression. Values with superscripts ‘^a,b,c,d^’ refers to significant (*p* < .05) differences in relative transcript abundance between groups

In assessment of abundance of transcripts encoding various motility‐ and fertility‐related genes in epididymal sperm, it was shown that transcripts encoding TSSK6 were found to be affected by freezing especially in slow progressive motility status (*p* < .01) and their relative expressions were higher in fresh than frozen‐thawed sperm (Figure 2).

There was no difference between fertility related genes (PRM1 and PRM2) in high motile fresh and frozen‐thawed sperm that were used in the IVF procedure. Freezing of sperm, however, decreased these fertility‐related genes especially in low motile sperm (*p* < .01; Figure 2).

As shown in Table 3, the cleavage rate did not present any significant differences between in vitro produced bovine embryos derived from fresh or frozen‐thawed epididymal sperm. Furthermore, the type of sperm, fresh or frozen‐thawed, had no effect on the overall blastocyst yield at days 7 (range 16.26%–18.05%) and 8 (range 21.77%–25.26%) among the two groups. No significant difference in each examined days (days 7 and 8) between the blastocysts rate of fresh and frozen‐thawed sperm groups indicated that the developmental timing of bovine embryos to the blastocyst stage was not affected by the freezing of sperm.

**TABLE 3:** Mean ± *SEM* of developmental rate of bovine IVP Embryos with fresh or frozen‐thawed epididymal sperm

Groups	Oocyte no.	Cleavage no. (%)	Blastocyst/cleavage no. (%)	
			Day 7	Day 8
Fresh sperm	349	259 (74.35 ± 5.64)	57 (16.26 ± 1.44)	76 (21.77 ± 2.30)
Frozen‐thawed sperm	217	176 (81.06 ± 2.38)	39 (18.07 ± 1.35)	55 (25.26 ± 2.56)

Note

Data represent *n* = 10 replicates.

## 4 DISCUSSION

In vitro production of embryo with cryopreserved recovery of the epididymal spermatozoa from dead animals is a useful tool to rescue genetic material that otherwise would be lost, either from highly productive livestock or endangered species. In this study, the spermatozoa from epididymis can be recovered with acceptable efficacy overnight after animal's death.

Cryopreservation processes are known as being damaging to the sperm cells due to changes during the biotechnological process, the toxic and osmotic stress depicted by exposure to cryoprotectants and the formation and dissolution of extracellular ice crystals (Januskauskas, Johannisson, & Rodriguez‐Martinez, 2003; Watson, 2000). In addition to sperm motility, the sperm motility pattern, fertility potential and the pattern of gene expression can be considered as the sperm quality parameter. In this study, the effects of cryopreservation of epididymal bovine spermatozoa on velocity and the pattern of sperm motility, fertility potential and the expression panel of some motility (*TSSK6*) and fertility (*PRM1* and *PRM2*)‐encoding genes viability pattern were also evaluated.

Reports in some ruminants such as bovine, goat and red deer indicate spermatozoa from epididymides are less tolerant to cryopreservation than spermatozoa from an ejaculate (Chaveiro, Cerqueira, Silva, Franco, & Moreira da Silva, 2015; Martins et al., 2009; Turri, Madeddu, Gliozzi, Gandini, & Pizzi, 2014; Zomborszky, Zubor, Toth, & Horn, 1999), especially when maintained in testes without blood flow and refrigerated during storage (Martins et al., 2009). After cryopreservation, the motility and its pattern were evaluated using CASA in the current study. As most studies have shown, cryopreservation of spermatozoa recovered from epididymides, caused a decrease in total motility of sperm, but no changes were detected in fast progressive motility. Few studies have analysed sperm collected from bovine epididymis using CASA (Goovaerts et al., 2006; Nichi et al., 2017; Reyes‐Moreno, Boilard, Sullivan, & Sirard, 2002). Goovaerts et al. (2006) collected sperm samples from bovine epididymis within five hours after slaughter, stored them at 4°C and subjected them to CASA (Goovaerts et al., 2006). Their reported CASA parameter values were comparable to those found in our and other studies (Nichi et al., 2017) on sperm samples collected from epididymis stored at 4°C for a few hours before cryopreservation. In this study, testicles were refrigerated for a longer period than on the mentioned studies (Goovaerts et al., 2006; Nichi et al., 2017), without suffering more severe deleterious effects after freezing.

In cryopreservation, beside the moderate reduction of motility, the frozen‐thawed epididymal sperm showed a more obvious decrease variables related to swimming velocity (VCL and VAP) with lower lateral displacements (MAD, ALH and BCF); whereas the linear trajectories of the sperm was not affected by cryopreservation (VSL, LIN, STR and WOB). In this study, the motility pattern of fresh and frozen‐thawed sperm reflects the lack of any statistical differences in the developmental rate of bovine embryo‐derived by fresh or frozen sperm, because the oocyte is probably fertilized with progressive sperm with lower lateral displacements (Jeulin et al., 1986). Also, the significant difference in the velocity of the frozen‐thawed sperm, which was seen only in slow progressive sperm, is justified by this reason, because those sperm with slow progressive motility have more lateral displacement that in the study was found in fresh sperm. Furthermore, cryopreservation negatively affected mitochondrial potential (Nichi et al., 2017). Since the mitochondria are the main source of energy in the most mammal's sperm, the motion parameters changes related to lateral displacements of the sperm is because of impaired mitochondria following cryopreservation (Hung, Miller, Meyers, & VandeVoort, 2008). Therefore, in our study, the reduction of ALH, and other motion parameters of sperm such as VCL, VAP, MAD and BCF, which can be dependent on mitochondrial activity is likely, due to the harmful effect of cryopreservation on sperm mitochondria.

Various experiments have been performed to predict sperm quality. In this regard, transcriptomic profiling of spermatozoa assumes importance as it carries information about spermatogenesis, sperm function and paternal roles in post‐fertilization events. Limited studies in the transcriptomic profile of bovine spermatozoa have shown that the spermatozoa contain various classes of RNA. In bovine, approximately 13,000 genes provide a series of spermatozoal transcripts, of which most are fragmented in nature. Cryopreservation communicates structural and molecular changes in the spermatozoa affecting sperm function (Selvaraju et al., 2018; Shilpa et al., 2017) compromising fertilizing ability.

Studies have recognized and quantified the sperm cryogenic changes characterised by sperm function and fertility alterations. Although the use of spermatozoal transcripts as markers of sperm quality and fertility (Jodar et al., 2016; Parthipan et al., 2017) has been developed, the studies focusing on spermatozoal transcripts and ultralow temperatures are limited even though most of the RNA studies emphasis on frozen specimens (Card, Krieger, Kaproth, & Sartini, 2017; Chen et al., 2015; Ganguly et al., 2013; Kopeika, Thornhill, & Khalaf, 2015; Zeng et al., 2014).

The composition and abundance of spermatozoal transcripts vary between fresh and frozen‐thawed bull sperm (Card et al., 2013; Selvaraju et al., 2018) probably due to degradation of mRNA during cryopreservation (Yeste, 2016) if appropriate measures are not implemented during vitrification/freezing or warming/thawing. For example, some studies showed that PRM1 (Ganguly et al., 2013) and DNMT3a and DNMT3B (Zeng et al., 2014) are affected during cryopreservation; exactly as we observed in our study about of given transcripts. In this study, abundance of *TSSK6* transcripts was lower in post‐frozen‐thawed sperm especially in low motile sperm and their relative expressions were higher in fresh than frozen‐thawed sperm. This is in accordance with a previous study showed that the transcripts encoding for a serine/threonine testis‐specific protein kinase (TSSK6) were related to high‐motility status in the bull and their relative expressions were higher in fresh than frozen‐thawed sperm (Mondal, Baruah, Chatterjee, & Ghosh, 2013). But we did not show any difference between the transcription levels of this gene in both low and high motile sperm, either before or after freezing that its cause is unclear to us. However, the effect of cryopreservation on reducing the level of these transcripts was significant.

In accordance with the developmental rate, no differences were found in relative expressions of PRM1 and PRM2 genes between high motile fresh and frozen‐thawed sperm that were used in IVF procedure. Freezing of sperm decreased these fertility‐related genes in low motile sperm. In bovine, though PRM1 gene has high transcription and translation levels, the PRM2 has been reported to be transcribed and translated at low levels in spermatids (Maier, Nussbaum, Domenjoud, Klemm, & Engel, 1990) if at all, but absent in spermatozoa (Hecht et al., 2011). The levels of PRM1 transcripts were significantly higher in good semen producers than poor counterparts, while the low transcription level of PRM2 did not differ between good and poor quality semen bulls (Ganguly et al., 2013). It should be emphasised that PRM1 and PRM2 genes are among the most strongly associated genes with different sperm quality parameters, such as morphology, motility and DNA integrity, as well as with fertility and finally embryo quality (Card et al., 2013, 2017; Dadoune, 2009; Ganguly et al., 2013). Evidence in this and other studies have been shown that the mRNA expression levels of PRM1 and PRM2 were significantly decreased in frozen‐thawed bovine sperm (Ganguly et al., 2013), thus emphasising the results of different studies, and indicating that sperm DNA damage is induced by cryopreservation process (Castro et al., 2016; Gurler et al., 2016; Kopeika et al., 2015; Martin, Sabido, Durand, & Levy, 2004).

Beside the above‐mentioned claims, the reduced levels of RNA in sperm cells during the cryopreservation may be due to changes in solution effect, ice crystal formation, free radicals, activation of the apoptotic and necrotic factors, pH and alteration in the membrane structure (Selvaraju et al., 2018). Although the mature spermatozoa are transcriptionally inactive (Grunewald, Paasch, Glander, & Anderegg, 2005), activation of the apoptotic factors during sperm cryopreservation (Martin et al., 2004) with the subsequent induction in expression of apoptosis‐related genes such as caspases genes have been reported (Paasch, Grunewald, Agarwal, & Glandera, 2004). However, the impact and mechanism of the cryo‐induced spermatozoal transcript profiles are not yet understood and accordingly, their influence on fertility and embryo developmental competency remains to be elucidated. Additional studies would help to optimize the cryopreservation protocol for maintaining and stabilising the sperm functional capacity.

Although IVF techniques have been widely studied in cattle, few studies were found regarding the evaluation the potential of in vitro fertilizing of epididymal spermatozoa from dead bulls (Chaveiro et al., 2015; Martins et al., 2009; Martins, Rumpf, Pereira, & Dode, 2007; Nichi et al., 2017). Regarding IVF results, developmental rate of in vitro produced embryos using frozen/thawed epididymal sperm showed no significant differences in comparison with fresh counterpart. In terms of cleavage and blastocyst rate, the values are similar or more than those obtained by some studies (Chaveiro et al., 2015; Martins et al., 2007).

Despite the prominent effects of cryopreservation on bovine sperm cells, in vitro derived embryos with recovered spermatozoa from the cauda epididymal of recently slaughtered animals showed that spermatozoa can be obtained from the epididymis of either a highly productive farm animals or endangered species, which can be used for the production of viable embryos. It seems epididymides with providing the optimal environment for sperm storage in physiological conditions have adequate conditions to prolong sperm survival.

In this study, the blastocyst rate was recorded in two days (day 7 and 8) as a simple marker for developmental timing. Timing of embryo development has long been considered as an important marker to evaluate embryo quality. The speed of embryo development can be used a simple, rapid, precise and noninvasive method of embryo selection for transfer (Sallam, Sallam, & Sallam, 2016). Although the developmental timing of in vivo produced embryo usually proceeds according to a precise program in all species, in vitro produced embryos commonly show heterogeneity in their developmental timing and subsequently variability in developmental competence (Gutierrez‐Adan, White, Van Soom, & Mann, 2015). The embryos with a timely manner development have the better developmental competence and viability after transfer, as shown in some studied species including human (Racowsky et al., 2000), rodents (McKiernan & Bavister, 1994) and cattle (Lonergan et al., 1999). Beyond the controversy surrounding the relation of developmental timing and embryo quality of bovine, no significant different blastocysts rate in each day (day 7 and 8) between the fresh and frozen‐thawed sperm groups indicated that the developmental timing of bovine embryos to the blastocyst stage was not affected by the freezing of sperm.

In conclusion, our results indicate that (a) epididymal sperm had enough fertilizing potential and freezability even after overnight testes storage; (b) cryopreservation could affect the relative abundance of transcripts available in the sperm cell, but the percentage of in vitro produced embryo was not much affected; (c) cryopreservation changed the sperm motility pattern further in the direction of progressive movement with less lateral displacement.

The present study showed the potential for utilization of dead animal epididymal sperm of for preservation of valuable males.

## CONFLICT OF INTEREST

None of the authors have any conflict of interest to declare.

## AUTHOR CONTRIBUTION

**Hassan Nazari:** Conceptualization; Data curation; Formal analysis; Funding acquisition; Investigation; Methodology; Project administration; Resources; Software; Supervision; Validation; Visualization; Writing‐original draft; Writing‐review & editing. **Ebrahim Ahmadi:** Formal analysis; Investigation; Methodology; Resources. **Hamid Hosseini Fahraji:** Resources; Software; Visualization. **Azita Afzali:** Methodology; Resources; Visualization; Writing‐original draft; Writing‐review & editing. **Najmeh Davoodian:** Formal analysis; Project administration; Writing‐original draft.

### PEER REVIEW

The peer review history for this article is available at https://publons.com/publon/10.1002/vms3.355.

## Data Availability

Data are available on request from the authors.
